# Supplementary material for: Essentiality of c-di-AMP in Bacillus subtilis: Bypassing mutations converge in potassium and glutamate homeostasis
Source: PLoS Genet. 2021 Jan 22;17(1):e1009092. doi: 10.1371/journal.pgen.1009092 (PMC7857571; doi:10.1371/journal.pgen.1009092)
Supplement: S1 Text — (DOCX) [file pgen.1009092.s001.docx]

**S1 Supporting Information**

**Global gene expression in the *B. subtilis* wild type strain in response to the potassium concentration and the nitrogen source.**

In order to study the consequences of changing environmental potassium and glutamate concentrations, we cultivated *B. subtilis* 168 in MSSM minimal medium with 0.1 or 5 mM KCl and with ammonium or glutamate as the nitrogen source, and analyzed the global transcriptomes (see S1 Table and S11 Fig for the data).

First, we considered the expression of the genes involved in c-di-AMP homeostasis (S2 Table). Of the three genes encoding the diadenylate cyclases, *cdaA* and *disA* were expressed under all tested conditions, whereas the *cdaS* gene was not expressed with ammonium as the nitrogen source or with glutamate at a low potassium concentration. Even in the presence of 5 mM potassium and glutamate, the expression of *cdaS* was very low as compared to the other cyclase-encoding genes. The expression of *cdaA* and *disA* was about three-fold reduced at the high potassium concentration if glutamate was present. In contrast, these genes were not affected by the potassium concentration when ammonium was the nitrogen source. The *gdpP* and *pgpH* genes that encode the two c-di-AMP-degrading phosphodiesterases were also expressed under all tested conditions. Again, the expression was reduced at 5 mM potassium if glutamate was present as the nitrogen source. The fact that the diadenylate cyclase and phosphodiesterase genes are regulated in the same manner, even though the encoded enzymes catalyze opposing reactions, suggests that the control is mainly exerted at the post-transcriptional level.

In the presence of glutamate, the *kimA* and *ktrAB* genes encoding high-affinity potassium transporters were 112- and 29-fold induced by potassium limitation, whereas the *ktrC* and *ktrD* genes encoding the low-affinity potassium transporter were not affected by the potassium concentration. With ammonium as the nitrogen source, the *kimA* and *ktrAB* genes were much less responsive to potassium availability (17- and 4-fold induction by potassium limitation, respectively). Interestingly, the expression of all potassium transporters is increased in the presence of glutamate if potassium is limiting in the medium. The expression of the *gltAB* operon encoding the glutamate synthase is 8- and 33-fold repressed by glutamate in the presence of 0.1 mM and 5 mM KCl, respectively. Importantly, in the absence of glutamate, the expression of the operon is fivefold enhanced at the increased potassium concentration. Thus, the gene expression data confirm the interrelation between potassium and glutamate homeostasis. To obtain independent evidence for this hypothesis, we studied the activities of the *kimA* and *gltAB* promoters using fusions of a promoterless *lacZ* gene encoding β-galactosidase to the corresponding promoter regions. For the *kimA-lacZ* fusion present in *B. subtilis* GP2181, we observed a strong repression at increasing KCl concentrations, as described earlier [1]. In addition, the expression of *kimA* was strictly dependent on the presence of glutamate, even at low potassium concentrations (see S12 Fig). The expression of the *gltA-lacZ* fusion was assayed using strain GP342. In the absence of glutamate, glutamate synthesis, and thus expression if the *gltAB* operon, is essential for the bacteria. However, the activity of the *gltA* promoter is increased from 300 to about 450 units per mg of protein, if the potassium concentration in the medium is increased from 0.1 mM to 5 or 20 mM KCl (S12 Fig). Thus, the reporter assays support the conclusion that the expression of potassium transporters is increased in the presence of glutamate whereas the expression of the *gltAB* operon is enhanced at high potassium concentrations. These results are in excellent agreement with the observed interdependence of the cellular potassium and glutamate pools, and provide an explanation for this mutual dependence of the two ions.

We also analysed the transcriptome data to identify the genes and operons that are most strongly affected by the availability of potassium and glutamate. In addition to the potassium transporters, genes of the Rex regulon that are involved in respiration and fermentation are strongly repressed by potassium (in particular in the absence of glutamate): the *cydABCD* and the *ldh-lctP* operons encoding the high-affinity terminal quinol oxidase and the lactate dehydrogenase are repressed 563 and 327-fold, at increased potassium concentrations (see S1 Table and S11 Fig). In contrast, most sporulation genes are switched off at limiting potassium concentrations suggesting an increased demand for potassium in spores. Finally, the genes of the Fur regulon that are normally induced upon iron starvation depend on potassium for their expression. The availability of glutamate, as expected, most drastically affected the genes of the TnrA regulon that are required for the acquisition of alternative nitrogen sources. These genes are strongly repressed by the preferred nitrogen source ammonium. Interestingly, the *tapA-sipW-tasA* and *epsA-O* operons encoding the major components of the extracellular biofilm matrix are both strongly dependent on the presence of glutamate, and the expression is further enhanced if sufficient potassium (5 mM KCl) is available. The expression of these biofilm genes is not known to be regulated by any of the known regulators involved in nitrogen or potassium regulation. Finally, the Rex and SigD regulons involved in respiration and motility, are strongly repressed by glutamate in the presence of 0.1 mM or 5 mM KCl, respectively.

**Reference**

1. Gundlach J, Herzberg C, Kaever V, Gunka K, Hoffmann T, Weiß M, et al. Control of potassium homeostasis is an essential function of the second messenger cyclic di-AMP in *Bacillus subtilis*. Sci Signal. 2017; 10: eaal3011.
