# Supplementary material for: Essentiality of c-di-AMP in Bacillus subtilis: Bypassing mutations converge in potassium and glutamate homeostasis
Source: PLoS Genet. 2021 Jan 22;17(1):e1009092. doi: 10.1371/journal.pgen.1009092 (PMC7857571; doi:10.1371/journal.pgen.1009092)
Supplement: S1 Table — (DOCX) [file pgen.1009092.s002.docx]

**S1 Table.**

**Transcriptomic data for the genes that are most strongly affected by the absence of c-di-AMP (Ammonium 0.1 mM KCl). ^1^**

| Gene Name | Wild type | | | | GP2222 | GP2223 |
| --- | --- | --- | --- | --- | --- | --- |
|  | Glutamate 0.1 mM KCl | Glutamate  5 mM KCl | Ammonium 0.1 mM KCl | Ammonium 5 mM KCl | Ammonium 0.1mM KCl | Ammonium 5 mM KCl |
| Upregulated in ∆*dac* | | | | | | |
| *abrB* | 2656 | 2945 | 1141 | 2853 | 10065 | 15703 |
| *ndhF-ybcC* | 52 | 29 | 106 | 46 | 716 | 56 |
| *nrgAB* | 94214 | 140635 | 335 | 406 | 2064 | 793 |
| *glnRA* | 23015 | 10532 | 2487 | 8233 | 14422 | 18522 |
| Downregulated in ∆*dac* | | | | | | |
| *yoaH* | 1149 | 124 | 944 | 2844 | 206 | 957 |
| *lytF* | 3981 | 838 | 4508 | 23400 | 975 | 9865 |
| *yfmG* | 1575 | 9329 | 1116 | 2588 | 232 | 1657 |
| *hag* | 79726 | 10341 | 89536 | 289197 | 18386 | 183228 |
| *ybdO* | 1735 | 672 | 1539 | 7172 | 315 | 3364 |
| *epr* | 6635 | 557 | 5952 | 25153 | 1216 | 11613 |
| *bmrU-bmr-bmrR* | 319 | 325 | 1177 | 166 | 237 | 197 |
| *yqbG* | 160 | 28 | 132 | 342 | 26 | 94 |
| *pgdS* | 10133 | 1310 | 7581 | 26097 | 1453 | 7445 |
| *yolAB* | 8079 | 2632 | 6905 | 32510 | 1307 | 13367 |
| *yorG* | 32 | 26 | 124 | 18 | 23 | 16 |
| *ydaC* | 50 | 36 | 420 | 19 | 79 | 20 |
| *ykuNOP* | 28 | 452 | 150 | 1076 | 28 | 608 |
| *tlpC* | 650 | 250 | 386 | 1920 | 69 | 572 |
| *hemAT* | 3071 | 1448 | 2749 | 13874 | 477 | 3405 |
| *yfmTS* | 6151 | 794 | 6869 | 22920 | 1135 | 8665 |
| *sspF* | 150 | 1951 | 6456 | 351 | 996 | 455 |
| *yonX* | 34 | 153 | 300 | 17 | 46 | 18 |
| *mcpC* | 2789 | 605 | 5106 | 16420 | 770 | 6978 |
| *yscB* | 863 | 139 | 1373 | 4568 | 181 | 2124 |
| *ywfA* | 3525 | 3510 | 911 | 45 | 120 | 54 |
| *yxbCD* | 5251 | 21069 | 6976 | 6617 | 905 | 10137 |
| *araE* | 41 | 50 | 660 | 19 | 76 | 35 |
| *yxbBA-yxnB-asnH-yxaM* | 4359 | 36716 | 8577 | 10124 | 962 | 14183 |
| *yxkH* | 429 | 68 | 286 | 2268 | 30 | 457 |
| *sivC* | 5987 | 2337 | 9378 | 40055 | 899 | 25992 |
| *yorE* | 32 | 28 | 237 | 21 | 21 | 18 |
| *yvrJ* | 26 | 26 | 189 | 18 | 15 | 18 |
| *ywcJ* | 60 | 75 | 2449 | 42 | 163 | 75 |
| *comGA---GG* | 19276 | 7644 | 545 | 294 | 31 | 63 |
| *yomK* | 758 | 71 | 880 | 1993 | 50 | 1324 |
| *sboAX* | 178 | 40 | 659 | 29 | 34 | 74 |
| *sigO-rsoA* | 65 | 46 | 661 | 70 | 28 | 97 |
| *tlpA-mcpA* | 668 | 141 | 911 | 1777 | 38 | 890 |
| *oxdC-rsiO* | 1274 | 644 | 11113 | 1152 | 339 | 577 |
| *cydABCD* | 73 | 39 | 12484 | 22 | 138 | 18 |
| *ldh-lctP* | 697 | 297 | 58791 | 180 | 1484 | 474 |

^1^ The numbers indicate the intensities as determined by the transcriptome analysis. For operons, data for the first gene are shown. The data are sorted according to the ratio of expression in medium containing ammonium and 0.1 mM potassium for the wild type 168 vs. the Δ*dac* strain GP2222.
