# Supplementary material for: Essentiality of c-di-AMP in Bacillus subtilis: Bypassing mutations converge in potassium and glutamate homeostasis
Source: PLoS Genet. 2021 Jan 22;17(1):e1009092. doi: 10.1371/journal.pgen.1009092 (PMC7857571; doi:10.1371/journal.pgen.1009092)
Supplement: S2 Table — (DOCX) [file pgen.1009092.s003.docx]

**S2 Table.**

**Transcriptomic data of genes involved in c-di-AMP, potassium, and glutamate homeostasis.** ^1^

| Gene name | Wild type | | | | Δ*dac* | |
| --- | --- | --- | --- | --- | --- | --- |
|  | Glutamate | | Ammonium | | Ammonium | |
|  | 0.1 mM KCl | 5 mM KCl | 0.1 mM KCl | 5 mM KCl | 0.1 mM KCl | 5 mM KCl |
| c-di-AMP homeostasis | | | | | | |
| *cdaA* | 10727 | 3014 | 6228 | 5878 | 18 | 20 |
| *disA* | 7600 | 2513 | 2492 | 3003 | 15 | 17 |
| *cdaS* | 26 | 621 | 30 | 19 | 17 | 19 |
| *pgpH* | 6932 | 2678 | 2860 | 3549 | 6659 | 4696 |
| *gdpP* | 4593 | 2199 | 6286 | 3623 | 6064 | 4994 |
| Potassium homeostasis | | | | | | |
| *kimA* | 98837 | 884 | 49463 | 2988 | 60648 | 7702 |
| *ktrA* | 6534 | 227 | 2462 | 645 | 8091 | 1662 |
| *ktrB* | 6425 | 301 | 1329 | 732 | 6573 | 1600 |
| *ktrC* | 3838 | 3294 | 2117 | 4643 | 5309 | 9220 |
| *ktrD* | 2729 | 1823 | 1248 | 1530 | 3246 | 2209 |
| Glutamate metabolism | | | | | | |
| *gltA* | 1529 | 2373 | 9269 | 59982 | 32726 | 62696 |
| *gltB* | 1917 | 2107 | 14529 | 69399 | 41312 | 71655 |
| *gltT* | 13499 | 6714 | 5335 | 12208 | 10594 | 12087 |
| *gltP* | 69 | 698 | 81 | 247 | 90 | 381 |
| *aimA* | 9882 | 1846 | 8135 | 10972 | 18750 | 17509 |

^1^ The numbers indicate the intensities as determined by the transcriptome analysis.
