# Supplementary material for: Essentiality of c-di-AMP in Bacillus subtilis: Bypassing mutations converge in potassium and glutamate homeostasis
Source: PLoS Genet. 2021 Jan 22;17(1):e1009092. doi: 10.1371/journal.pgen.1009092 (PMC7857571; doi:10.1371/journal.pgen.1009092)
Supplement: S3 Table — (DOCX) [file pgen.1009092.s004.docx]

**S3 Table.**

**Oligonucleotides used for qRT-PCR analyses.**

| **Name** | **Sequence** | **Gene** |
| --- | --- | --- |
| LK427 | ATGTATTTGGCGCTCCGCTTG | fwd_*cydA* |
| LK428 | CGTTCCGAATGATACGAGCCA | rev_*cydA* |
| LK431 | GCGCTGTCATCTGTCGCATA | fwd_*kimA* |
| LK432 | GCCCTGCGGATAAGCGTAAA | rev_*kimA* |
| LK433 | GGTCAATGCATATGCTTCTTACG | fwd_*ktrA* |
| LK434 | AATAGGGTGGTCAGCGTACTC | rev_*ktrA* |
| LK435 | GCGATGAGTCTTGCTCGCAT | fwd_*ldh* |
| LK436 | CAGCTCAGTGATACCTGCGA | rev_*ldh* |
