# Supplementary figures and images for: Essentiality of c-di-AMP in Bacillus subtilis: Bypassing mutations converge in potassium and glutamate homeostasis
Source: PLoS Genet. 2021 Jan 22;17(1):e1009092. doi: 10.1371/journal.pgen.1009092 (PMC7857571; doi:10.1371/journal.pgen.1009092)

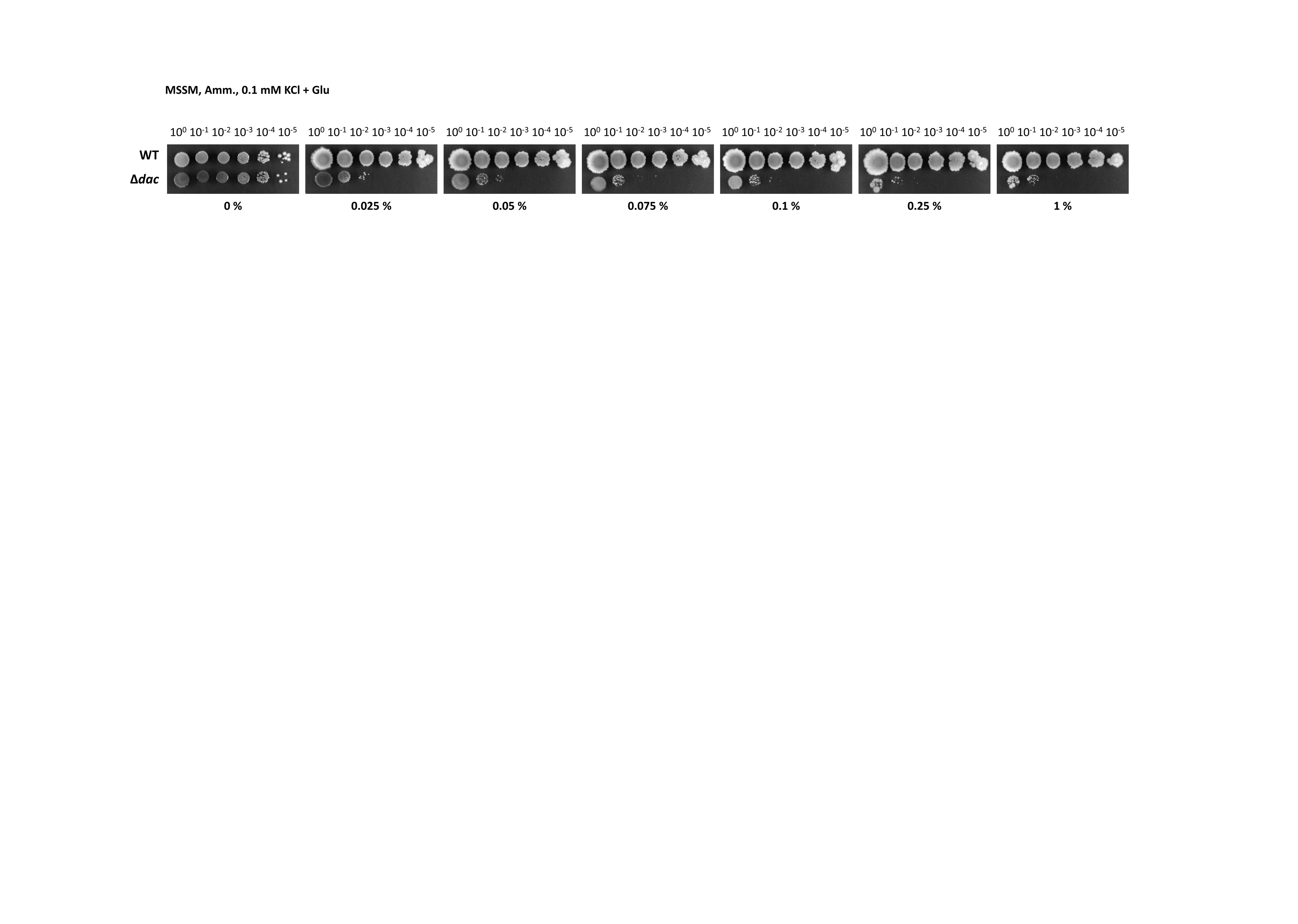

Supplement: S1 Fig — Growth of B. subtilis 168 and GP2222 (Δdac) was assessed on MSSM medium with ammonium and 0.1 mM and the indicated amount of glutamate (%). (TIF) [file pgen.1009092.s006.tif]

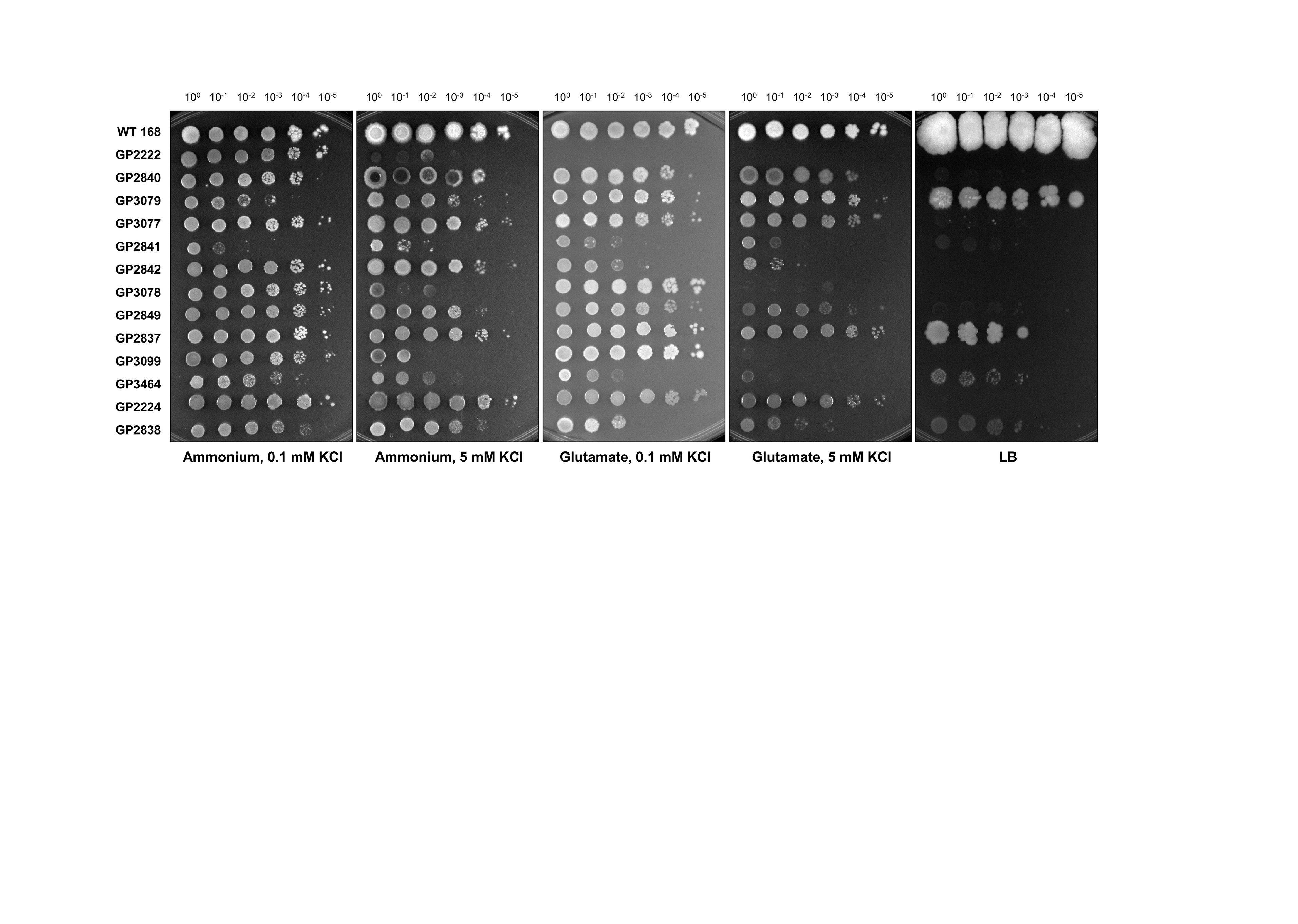

Supplement: S2 Fig — Growth assay of B. subtilis wild type, GP2222 (Δdac), and the isolated glutamate suppressor mutants. B. subtilis strains were cultivated in MSSM minimal medium with 0.1 mM KCl and ammonium. The cells were harvested, washed, and the OD600 was adjusted to 1.0. Serial dilutions were dropped onto MSSM minimal plates with the indicated potassium concentration and ammonium or glutamate, or on LB plates. (TIF) [file pgen.1009092.s007.tif]

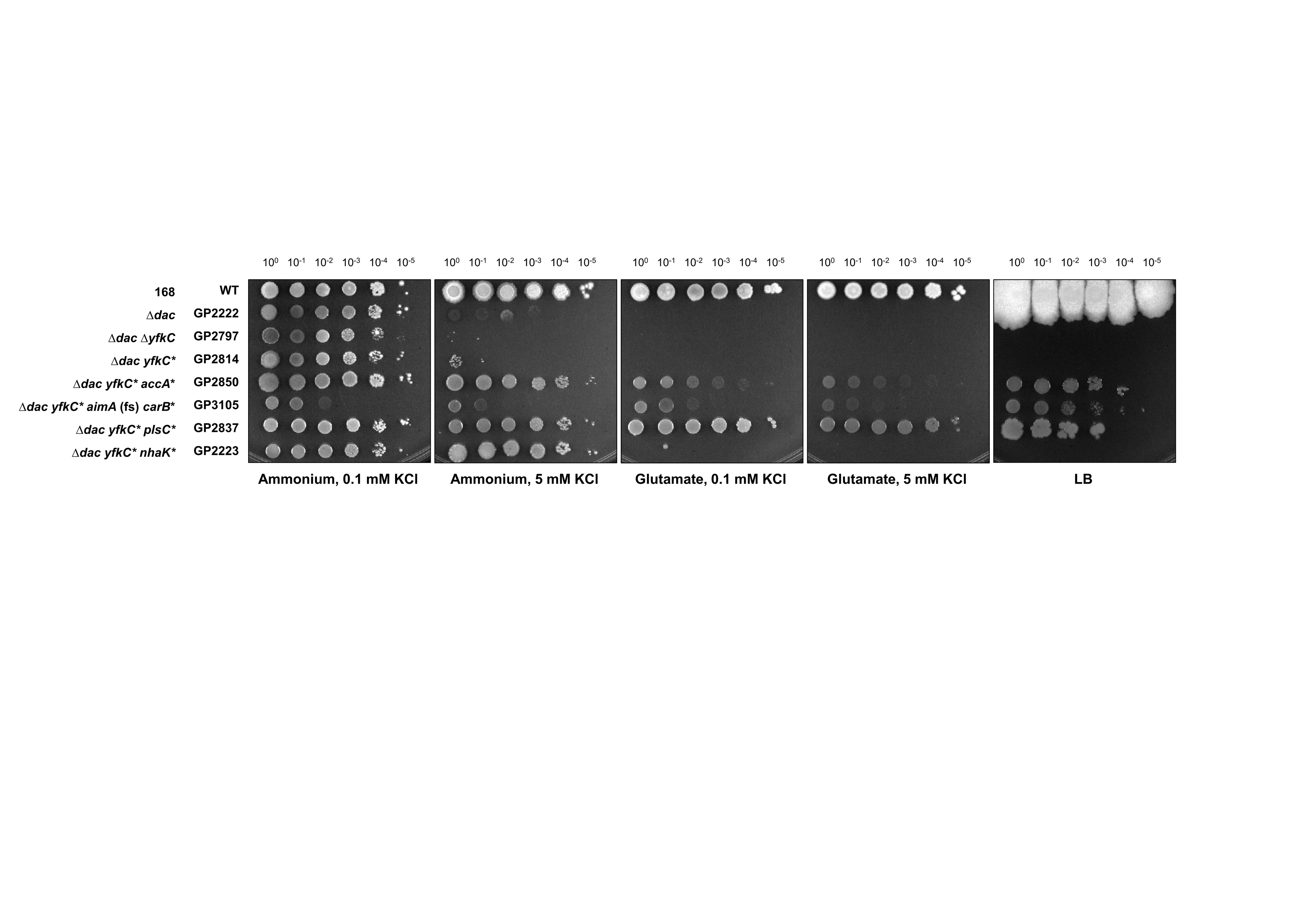

Supplement: S3 Fig — Growth assay of B. subtilis wild type, GP2779 (Δdac ΔdarB), GP2420 (Δdac ΔdarA) and the isolated glutamate suppressor mutants. B. subtilis strains were precultivated in LB medium. The cells were harvested, washed, and the OD600 was adjusted to 1.0. Serial dilutions were dropped onto MSSM minimal plates with 0.1 mM KCl and ammonium or on LB plates. (TIF) [file pgen.1009092.s008.tif]

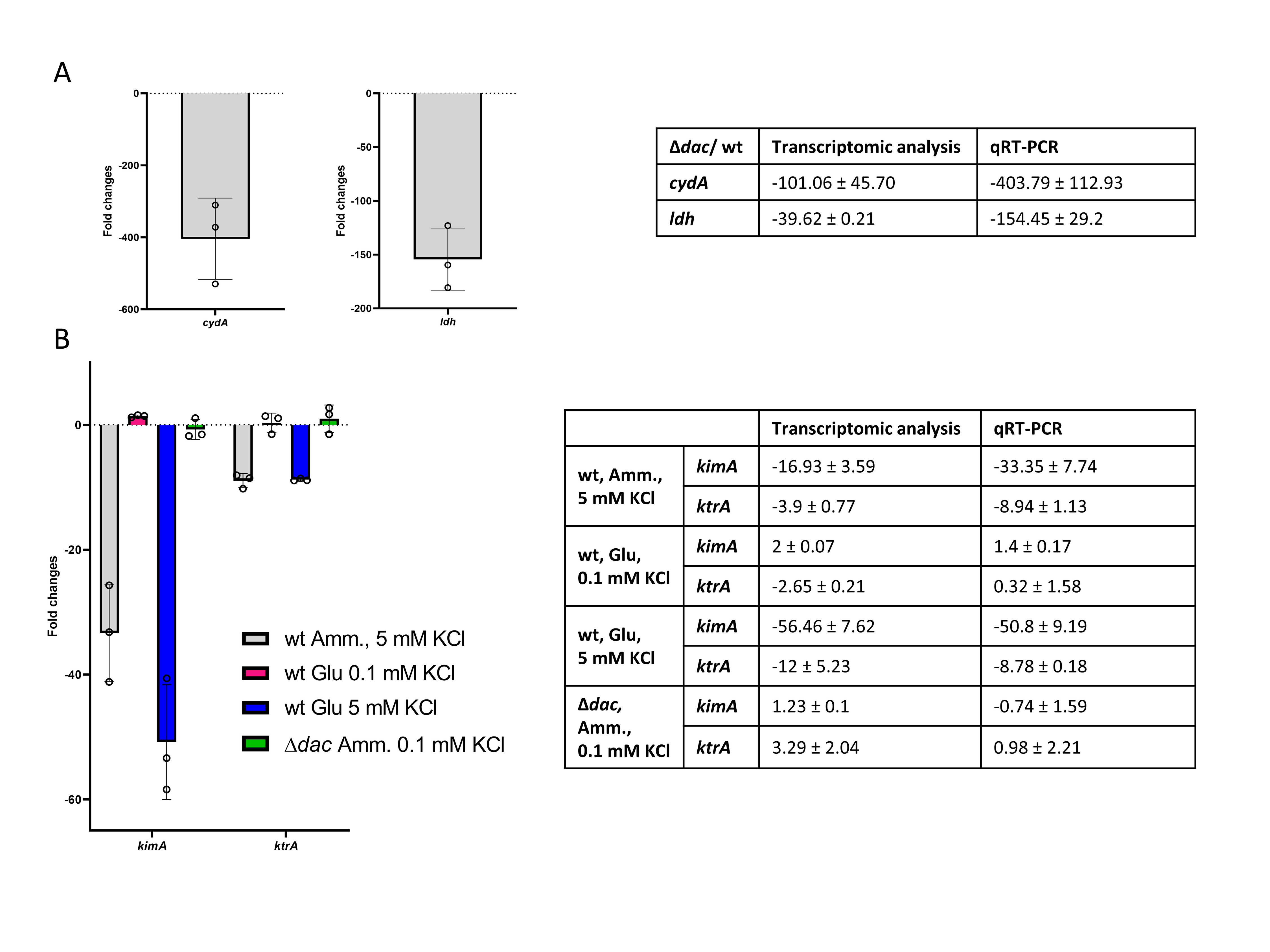

Supplement: S4 Fig — Validation of the c-di-AMP dependent regulation of genes involved in respiration and fermentation (A) and potassium uptake (B). B. subtilis wild type and Δdac were cultivated in MSSM medium with ammonium and 0.1 mM KCl (A) or in MSSM medium with ammonium or glutamate and 0.1 mM or 5 mM KCl (B) and RNA was extracted and quantified by qRT-PCR. Changes in expression are represented as fold changes compared to the wild type grown with Ammonium and 0.1 mM KCl. The tables show a comparison of the ratios as determined by the transcriptomic analysis and qRT-PCR. (TIF) [file pgen.1009092.s009.tif]

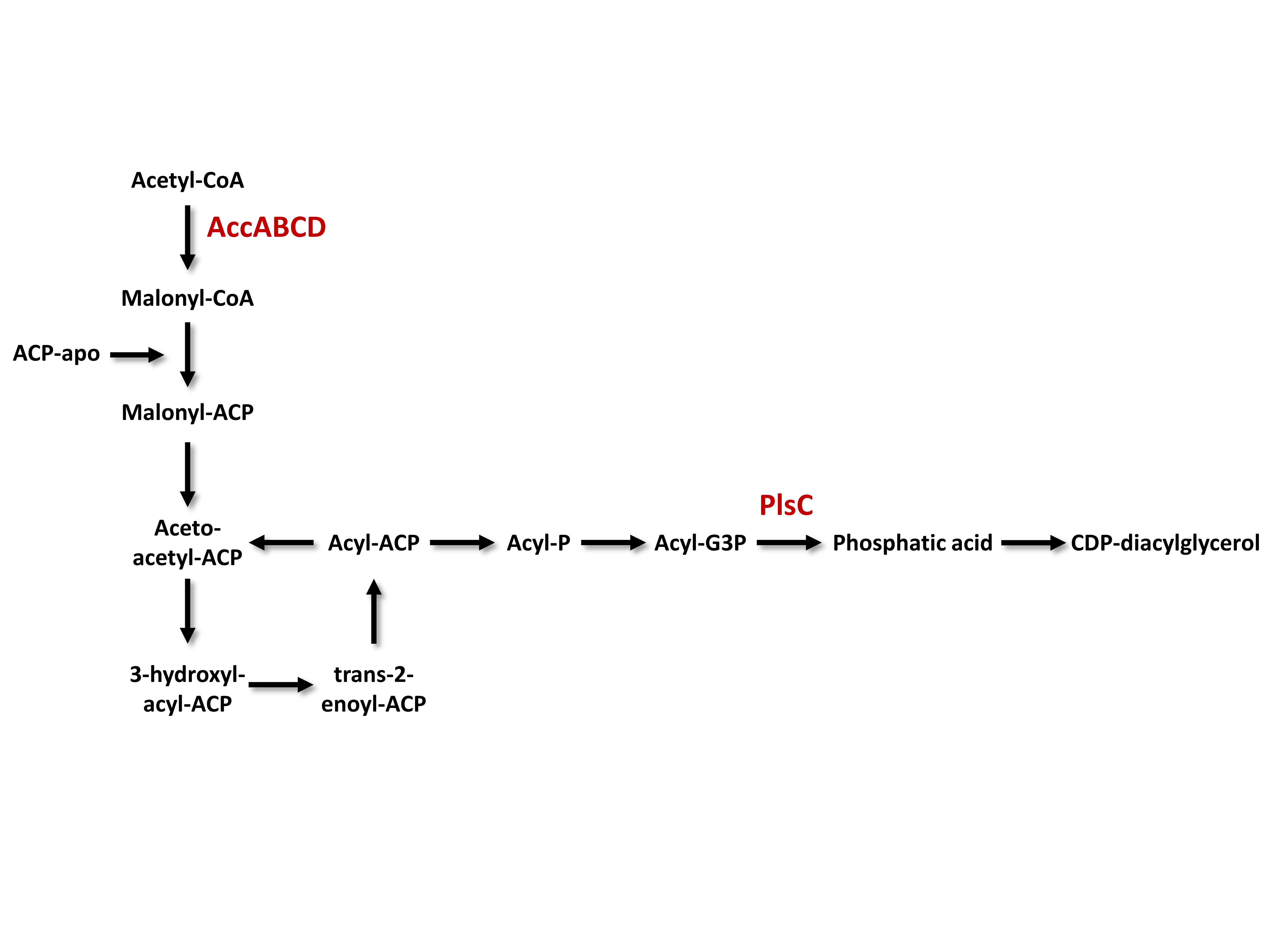

Supplement: S5 Fig — In the suppressor screen with glutamate (Table 1) and LB (Fig 2), mutations in accA, accC, and plsC, which are involved in the synthesis of phospholipids, were obtained. All mutations were single amino acid substitutions from independently isolated clones. All mutations are located in highly conserved regions of AccA and AccC (Acetyl-CoA carboxylase, S7 Fig) and PlsC (acyl-ACP:1-acylglycerolphosphate acyltransferase, S8 Fig) close to the active center. (TIF) [file pgen.1009092.s010.tif]

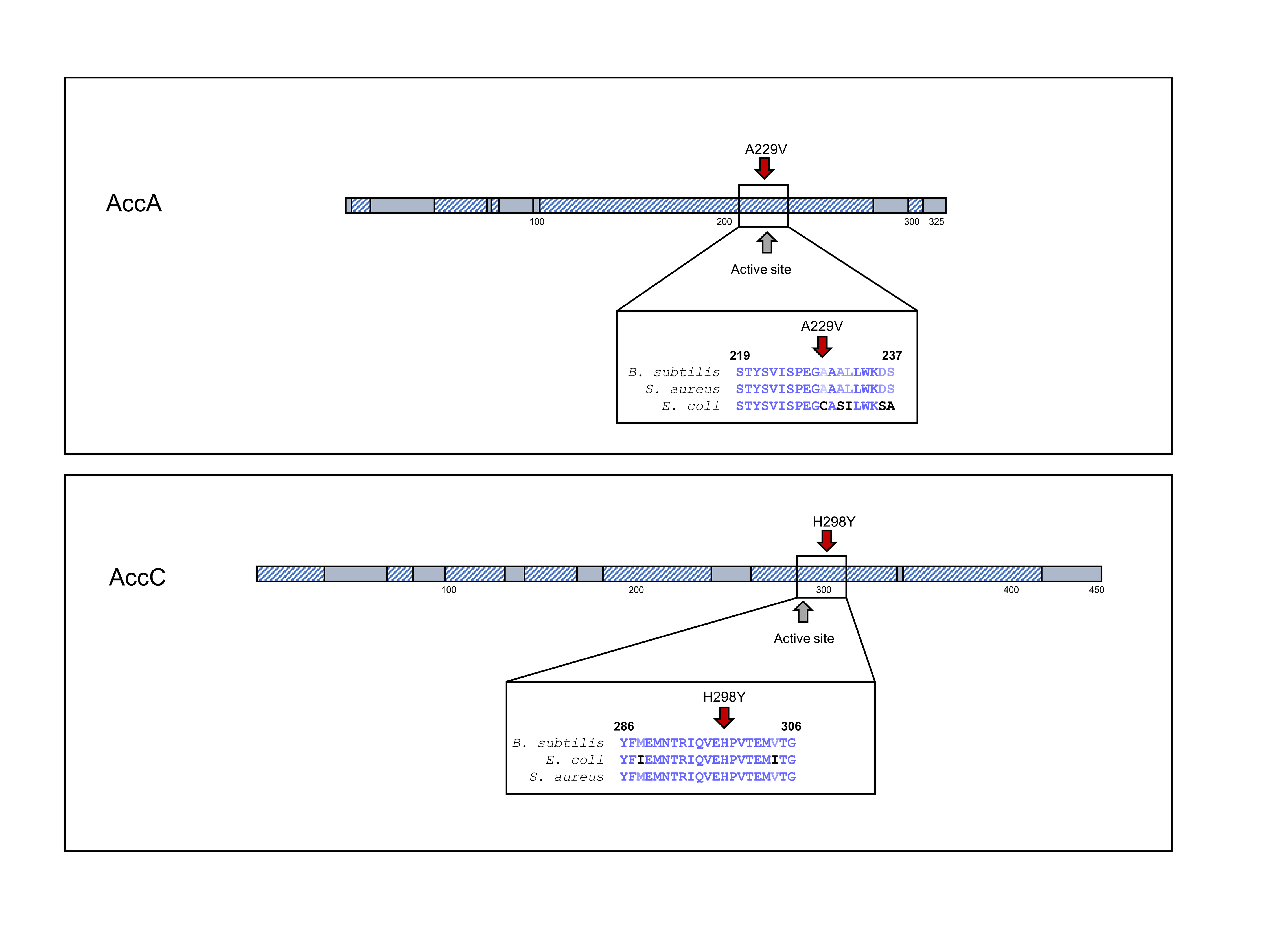

Supplement: S6 Fig — Mutations obtained in the suppressor screen with glutamate (Table 1) and LB (Fig 2). All mutations were single amino acid substitutions from independently isolated clones. Both mutations are located in highly conserved regions of AccA and AccC (hatched boxes) close to the active center. (TIF) [file pgen.1009092.s011.tif]

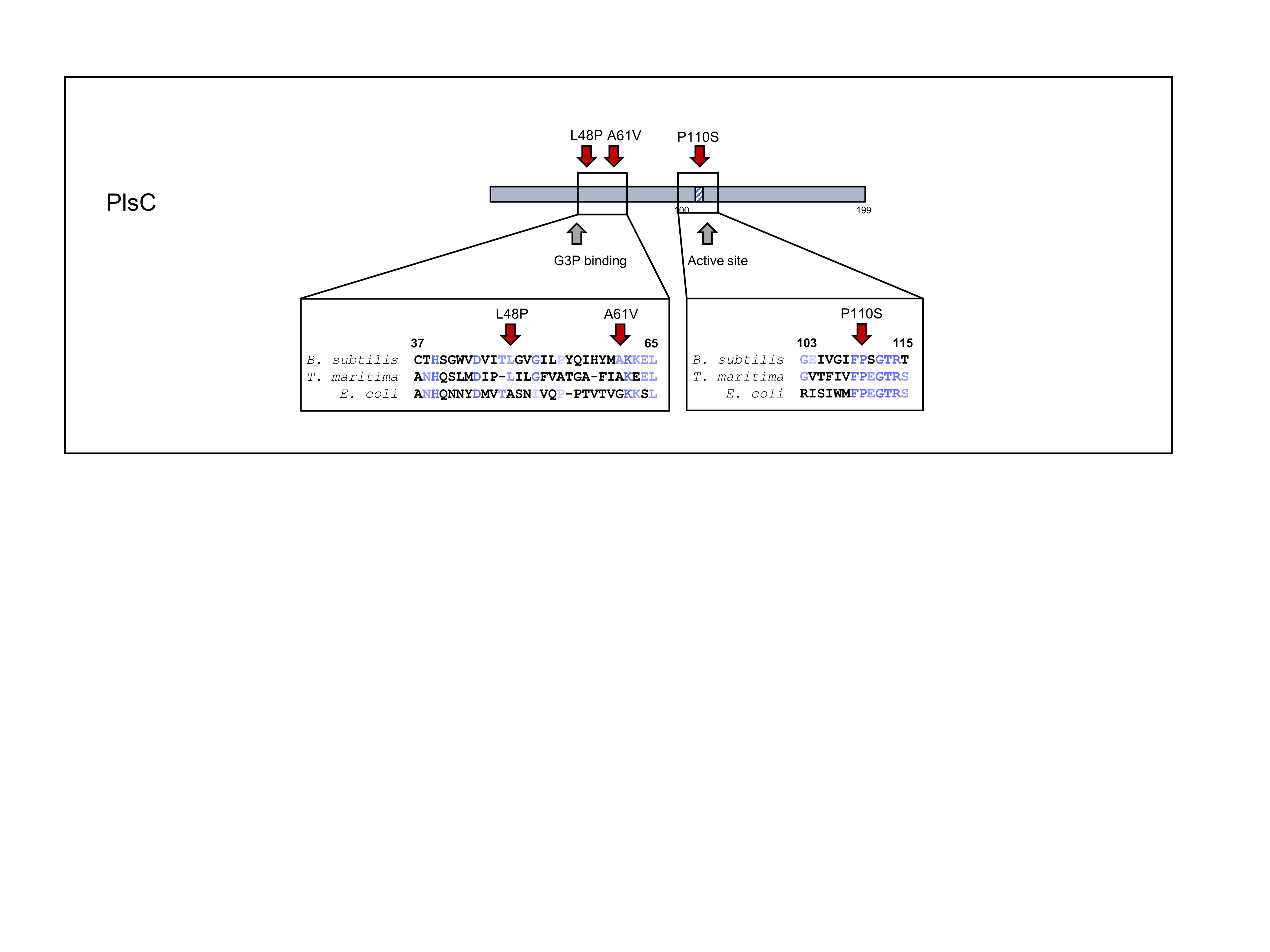

Supplement: S7 Fig — Mutations in the plsC gene were obtained in the suppressor screen with glutamate (Table 1). All mutations were single amino acid substitutions from independently isolated clones. All mutations are located in functionally important regions of the enzyme. (TIF) [file pgen.1009092.s012.tif]

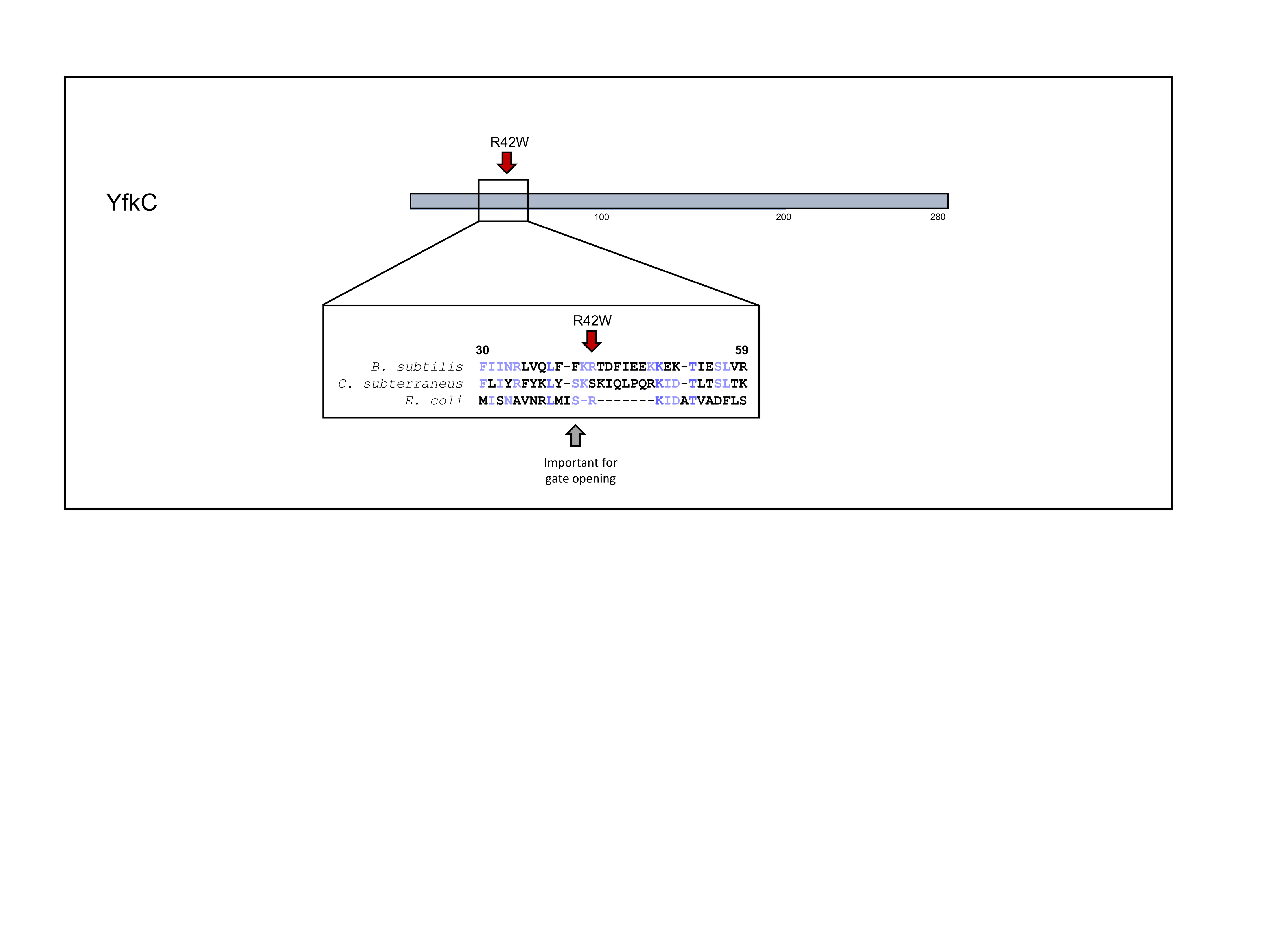

Supplement: S8 Fig — Mutations in the yfkC gene were obtained in the suppressor screen with glutamate (Table 1). The amino acid substitution Arg42 to Trp was observed in three independently isolated clones. In E. coli localization of S58 was observed to be highly dependent on the closing state of the channel, thus, it appears likely that amino acid residue 42 in B. subtilis is located close to the gate. (TIF) [file pgen.1009092.s013.tif]

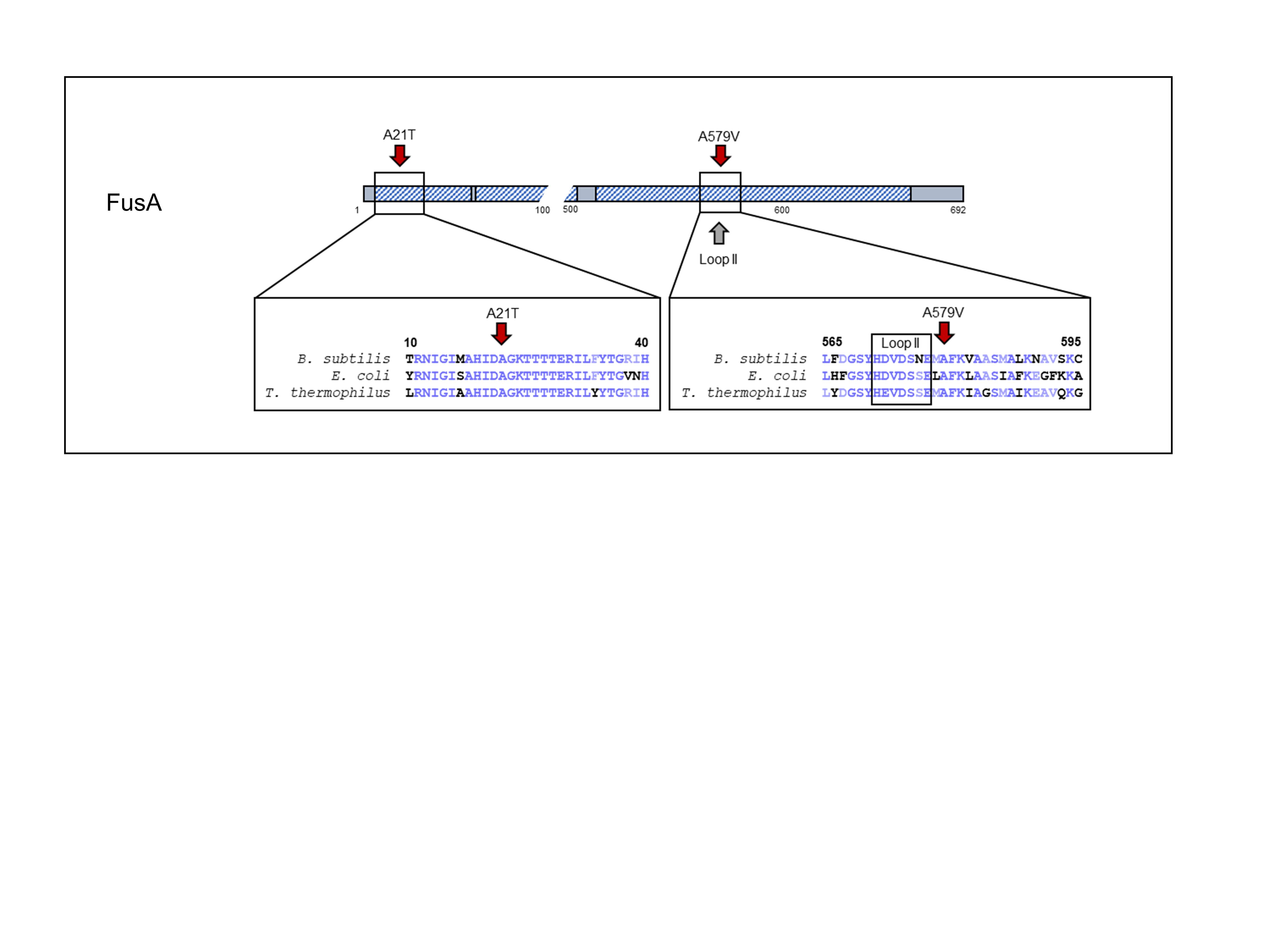

Supplement: S9 Fig — Mutations in the fusA gene were obtained in the suppressor screen of GP3054 (Δdac ΔaimA) with glutamate (Fig 1). The amino acid substitution Ala21 to Thr was observed in one suppressor. Ala21 is adjacent to Asp20, an amino acid residue that binds the Mg ion and is therefore crucial for GTPase activity of the protein. In the two other suppressors Ala579 was mutated to Val. This amino acid residue is located in the conserved loop II that is required for proper translocation. (TIF) [file pgen.1009092.s014.tif]

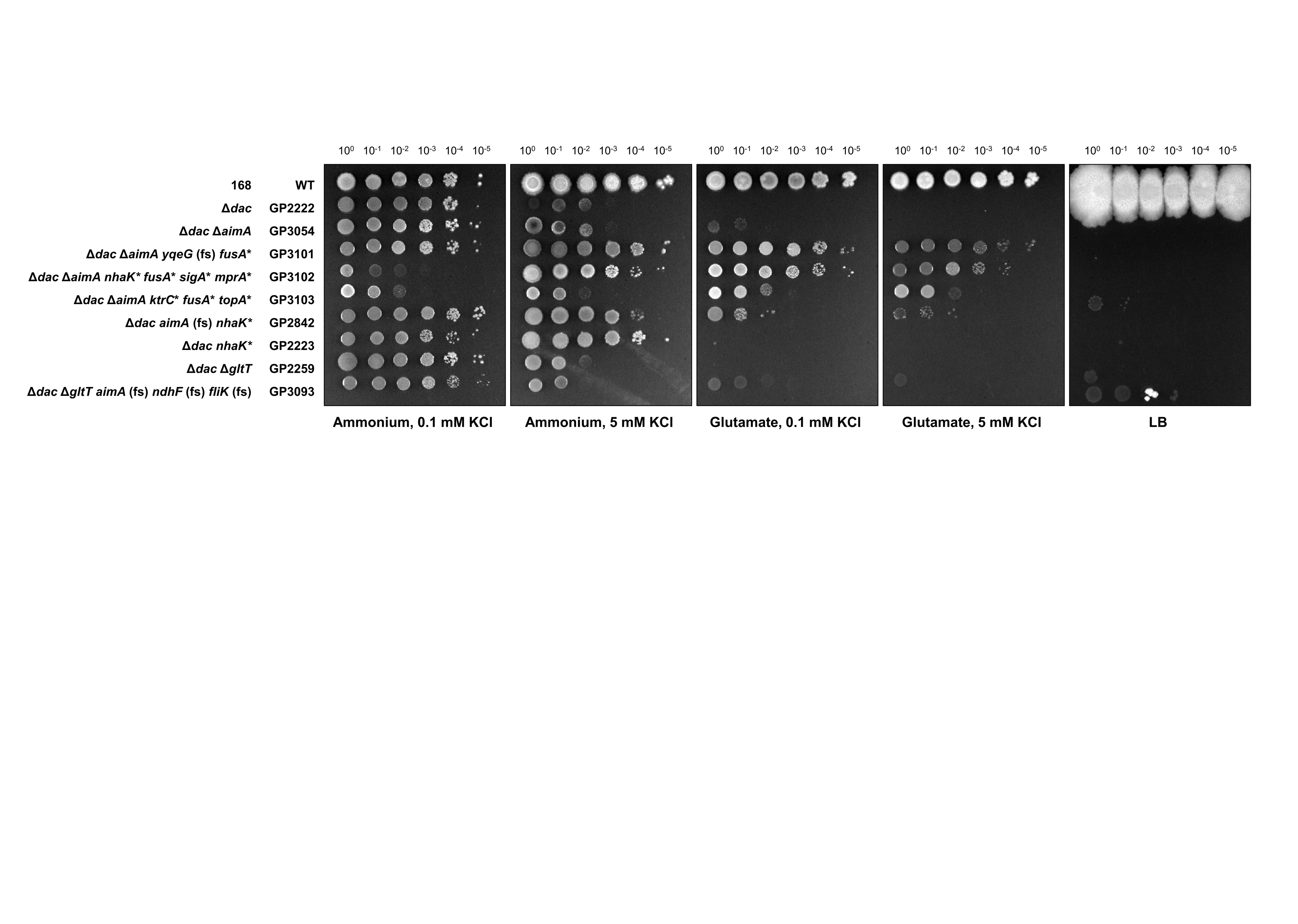

Supplement: S10 Fig — Growth assay of B. subtilis wild type, GP2222 (Δdac), GP3054 (Δdac ΔaimA), GP2248 (Δdac ΔgltT) and the isolated glutamate suppressor mutants. B. subtilis strains were cultivated in MSSM minimal medium with 0.1 mM KCl and ammonium. The cells were harvested, washed, and the OD600 was adjusted to 1.0. Serial dilutions were dropped onto MSSM minimal plates with the indicated potassium concentration and ammonium or glutamate, or on LB plates. (TIF) [file pgen.1009092.s015.tif]

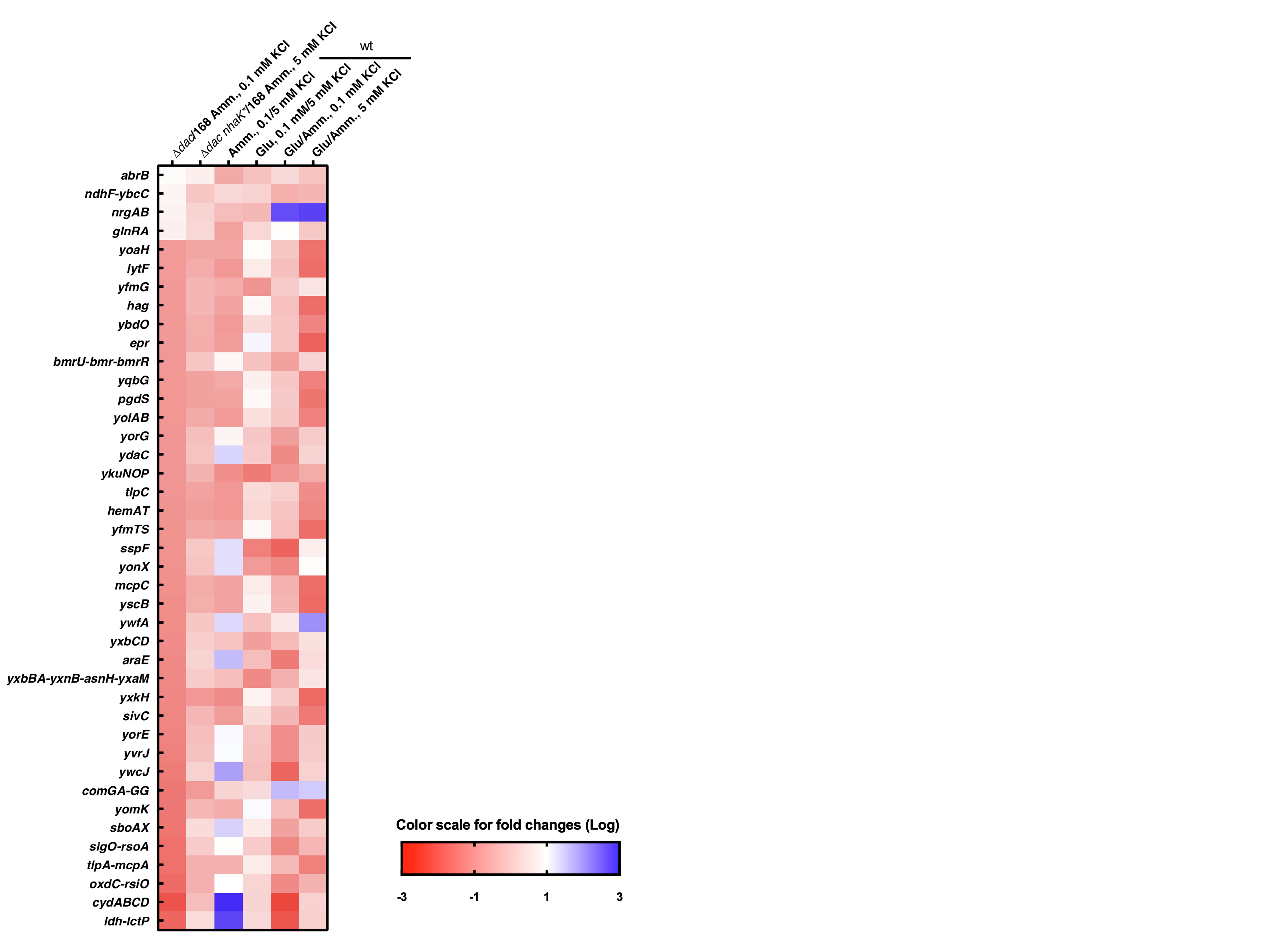

Supplement: S11 Fig — Representation of expression patterns of the most differentially expressed genes without c-di-AMP (see S1 Table). Blue and red indicate up- and downregulated expression, respectively. Color density represents the level of fold change. The figure was prepared with GraphPad Prism 8. (TIF) [file pgen.1009092.s016.tif]

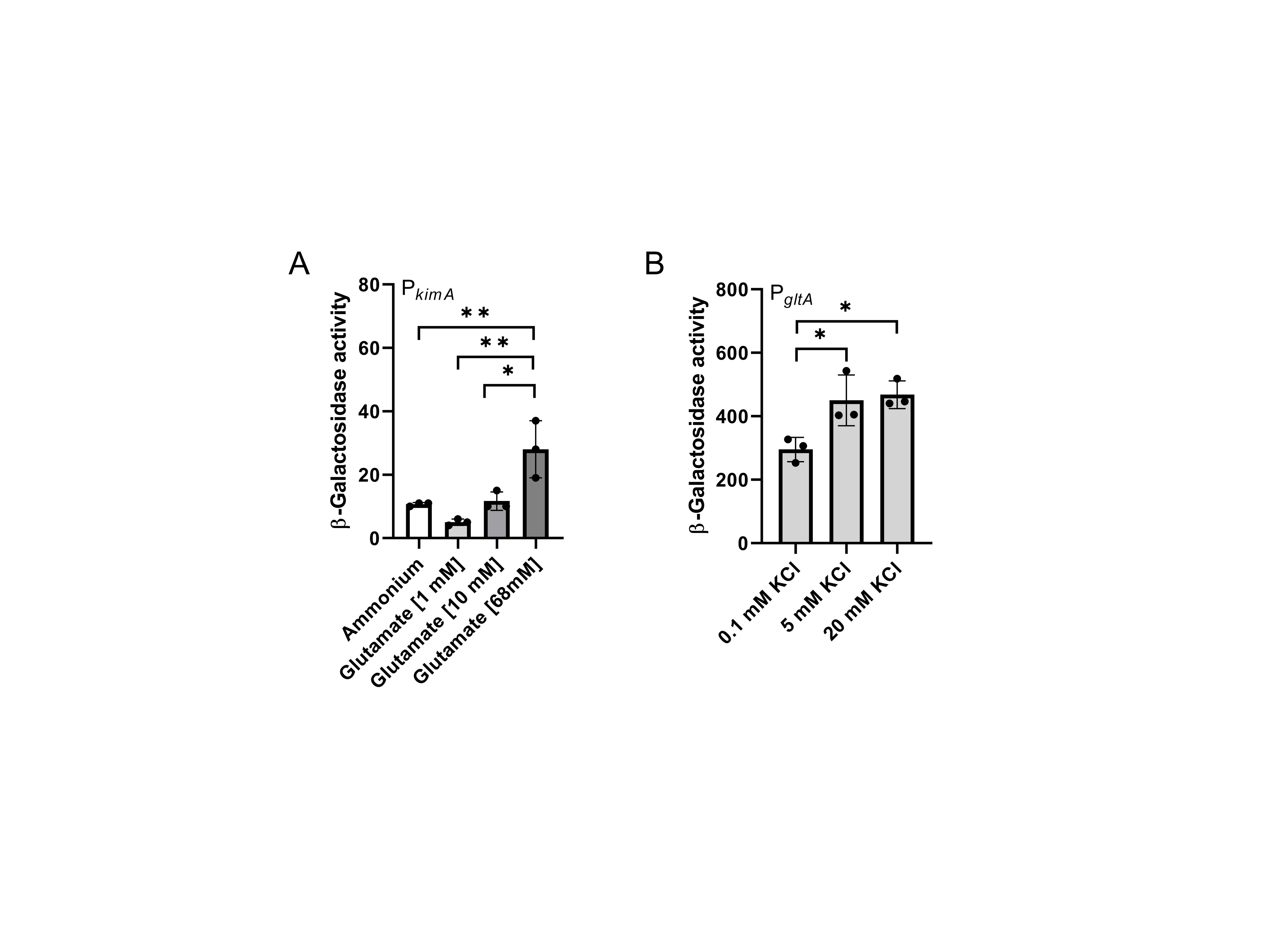

Supplement: S12 Fig — The expression of the high-affinity potassium transporter kimA (A) and the glutamate synthase gltA (B) was assessed by fusion of the promoter to the reporter gene lacZ. The cells harboring the promoter-fusion were cultivated with ammonium or glutamate at 0.1 mM KCl (kimA) or ammonium and different potassium concentrations (0.1, 5, 20 mM; gltA). Promoter activity was analyzed by quantification of β-galactosidase activity. Statistical analysis was performed using a one-way ANOVA, followed by Tukey’s multiple comparisons test (**** P < 0.0001). (TIF) [file pgen.1009092.s017.tif]
