# Supplementary material for: Essentiality of c-di-AMP in Bacillus subtilis: Bypassing mutations converge in potassium and glutamate homeostasis
Source: PLoS Genet. 2021 Jan 22;17(1):e1009092. doi: 10.1371/journal.pgen.1009092 (PMC7857571; doi:10.1371/journal.pgen.1009092)
Supplement: Attachment — Submitted filename: Rebuttal cda suppr.docx [file pgen.1009092.s018.docx]

**Comments to the Authors:**

**Reviewer #1:**

 c-di-AMP is an essential signaling molecule under standard lab growth conditions, although certain species lacking c-di-AMP can grow when plated on minimal media or when grown on complex media anaerobically. Previous work on this nucleotide has enabled the isolation of suppressor mutants that improve growth, with mutations affecting osmolyte or glutamate uptake. Here the authors do in-depth suppressor screening to uncover more about the importance of c-di-AMP for balancing potassium and glutamate homeostasis. Suppressor mutants are isolated on minimal media with toxic levels of glutamate or on complex media, both anaerobically and then aerobically. The authors conclude that the adaptation to these growth conditions requires the acquisition of multiple mutations, which frequently affect potassium, osmolyte or glutamate metabolism, but also find a connection to phospholipid biosynthesis. This manuscript showcases a lot of work that will certainly add to the field, and as such deserves publication. I have two recommendations that I feel would strengthen the manuscript and then some minor comments.

RE: We are grateful for these kind words about our work!

Major
• On line 184 the authors state “it seems likely that more than one mutation is required to achieve suppression”. In the methods it explains that the whole genome sequencing was performed on suppressors that had been re-isolated 3 times after the original colony appeared. Therefore it is currently not clear whether all of the mutations in Table 1 arose in the original suppressor colony or whether they were introduced during re-isolation to improve growth. This is something I remember reading about in Corrigan et al 2011 Plos Pathogens paper on c-di-AMP. An easy way to confirm the need for multiple mutations for suppression is to perform PCR on chromosome from the original colony to confirm that all mutations are present. It may also highlight a smaller number of mutations that are the most critical for suppression of the lack of c-di-AMP.

RE: We are sorry that the statement in Methods was not sufficiently clear. Actually, the suppressor mutants were re-streaked from the original colony three times to obtain a pure colony for subsequent work. This is now specified in the method section. This is the standard routine that we follow when studying suppressor mutants. The reviewer is correct in saying that we cannot exclude the acquisition of more mutations in the second or third “re-streak”; however, we feel that this is not so relevant since we look for strains that are best adapted to growth under the selective condition. Since we do store only the pure colonies obtained in the described way, we are not able to do a PCR on the initial colonies.

• Fig 4 shows a strong growth phenotype with suppressor strain GP2837, due to mutations in PlsC and YfkC. However the single YfkC* mutation does not improve growth, whereas the combination of YfkC* and PlsC L48P restores to near WT. Has the PlsC L48P mutation been tested alone? It may be that PlsC L48P is responsible for the restoration in growth and plays a larger role than YfkC*.

RE: This is a question that we also considered. However, even with repeated attempts, it was not possible to obtain a plsC-L48P single mutant suggesting that this mutation is not tolerated by the wild type strain. Anyway, the question of the reviewer draw our interest back to this issue. We therefore checked whether we could introduce the plsC-L48P mutation into a strain already carrying the yfkC* allele (otherwise wild type), and this was indeed possible. So it is clear that the yfkC* plsC* double mutant is viable whereas the plsC* strain is not (in the 168 background). We were not able to introduce the plsC* allele alone into the Δdac strain since this would require the initial construction of a plsC* strain still producing c-di-AMP which was not possible. It was not possible to introduce the allele into the Δdac strain because this strain cannot be transformed. However, based on the measurements of intra- and extracellular glutamate concentrations, that we have performed now and included in the manuscript (Fig. 4B, 4C), we think that the yfkC* mutation is decisive as it allows to reduce the intracellular glutamate concentration and concomitantly increase the extracellular concentration of glutamate, and this works best in combination with the plsC* mutation (as hypothesized in the original submission and demonstrated experimentally in the revised paper, see major comment of reviewer #3).

Minor
• The introduction, although quite long, should introduce the B. subtilis target proteins in greater detail. For example, KtrC is mentioned frequently in the results and should be introduced as the homologue of the KtrA system. DarA and DarB are introduced as “signal transduction proteins” but this is not very informative. One of two sentences on the targets would aid the reader.

RE: We have added some information on the major target proteins (l. 131 - 141).

• Line 641: what was the rationale behind incubating at 42^o^C to isolate suppressors? Wouldn’t this just stress the bacteria more?

RE: This was done since *B. subtilis* grows better at 42°C than at 37°C!

• Please use a footnote for Table 1 to define the scoring system.

RE: This has been added to footnote 1.

• Using a heat map for Table S1 would really help in visualizing what has gone up or down.

RE: We agree and have added the heat map as supplementary Figure S11.

• Line 178 – NhaK – what is this protein and how does it affect K+ homeostasis?

RE: We have specified this in the sentence that just follows: “Interestingly, the same mutation in the NhaK cation:proton antiporter NhaK (S187F) had been isolated in a suppressor screen of the Δ*dac* strain in the presence of 5 mM potassium [21] indicating that the mutant protein has an increased activity in potassium export. ”

• Line 376 and 377 – this statement refers to Fig S4, not S5. Similarly line 387 should refer to Fig S5, not S6.

RE: Thank you for drawing our attention to this issue, actually, we had to re-order the figures. Now, Figure numbers and references to them in the text match.

• Are there error bars and stats for Fig 5e? Please state in all of the legends how many repeats were performed and include statistics were appropriate.

RE: This is now included. The number of repeats is stated in the methods (see l. 670, 728, 734).

**Reviewer #2:**

The manuscript by Kruger et al. examined the Bacillus subtilis dac mutant, deleted for all three diadenylate cyclases and therefore devoid of c-di-AMP, to elucidate the mechanisms of glutamate toxicity to this mutant and the mechanisms of c-di-AMP essentiality in Bacillus growth in rich media (LB). Through multiple genetic screens, the authors isolated many suppressor mutants for each phenotype, and presented thoughtful analyses of each mutation. The identification of these mutations is useful to understand c-di-AMP signaling. The mechanisms for glutamate toxicity appear largely related to potassium homeostasis. The mechanisms for c-di-AMP essentiality in LB are unclear. There are some instances where a few more control experiments would further support the conclusions, and the clarity of the manuscript could be improved with some re-organization and provision of model figures.

RE: We are grateful to this reviewer for the helpful suggestions that, as we think, helped to improve the manuscript.

My scientific comments are as follows:

1. Although the suppressor screens revealed very interesting mutations, many remain to be validated, with a notable example of plsC mutations. If reduced phospholipid synthesis suppresses glutamate toxicity as the authors interpreted, can this phenotype be reversed upon expression of WT plsC in the relevant suppressor mutants? Similar complementation should also be feasible for other loss-of-function mutations, such as ndhF, fliP, etc.

RE: Unfortunately, the Δdac mutant is not genetically competent; therefore we were not able to construct such strains. Resulting from the reviewer’s request, we checked whether the suppressor mutants had regained genetic competence; however, this was not the case.

2. Related to my comment #1 above, I suggest that the transcriptomic data be validated by qPCR or transcriptional fusion reporter assays, at least for a select set of genes that the authors discussed in great details or were presented in the main figures, ie. gltA/aimA/gltP expression (Figure 5E), ndhF, ldh.

RE: We have validated the regulation of respiration/fermentation genes by qRT-PCR. This is now provided as Fig. S4. For glutamate transporters, the regulation was much less pronounced, and therefore difficult to verify by qRT-PCR.

3. What is the approximate glutamate minimal inhibitory concentration in the dac mutant? The suppressor screen for glutamate toxicity was performed with 1% glutamate (equivalent to 70 mM?), which seemed rather high considering that later experiments for glutamate growth (shown in Figure 5) were done for 20mM glutamate or less? On a related note, perhaps glutamate concentrations used would affect which glutamate transporters would be inactivated to suppress glutamate toxicity?

RE: We have tested the growth of the Δdac mutant at different glutamate concentrations, and we observe faint growth at 0.025% glutamate, whereas growth was nearly completely inhibited at 0.05% (with formation of suppressor mutants). This information is now described in the text (l. 164 - 168) and included as new supplemental Figure (S1 Fig.). And yes, the glutamate concentration may affect the choice of transporters to be inactivated.

4. The screen for dac suppressors in LB revealed several interesting phenotypes, but also somewhat complicated the interpretation of glutamate toxicity suppressors isolated from MSSM.

- Some glutamate resistant mutants in Table 1 grew in LB. Was growth observed under aerobic or anaerobic condition? If these mutants can grow aerobically in LB, how do the authors reconcile this observation with the LB suppressor screen results and discussion related to Figure 2?

RE: This is a good point. Growth was tested aerobically, and it is really difficult to put this into context with the LB suppressor screen!

- Was it surprising that glutamate transporters were not identified in the LB suppressor screen? What’s the glutamate content in LB?

RE: The glutamate concentration in LB is 11.2 mM. In addition, there is also potassium in LB. The precise concentration is not known to us, but taking into account that potassium and glutamate are the by far predominant ions in each living cell (including yeast), and that potassium even exceeds the glutamate concentration, we can assume, that the potassium concentration in LB is in the same range as the glutamate concentration. And yes, it was surprising that none of the LB suppressors carried mutations specifically related to glutamate. One possibility would be that DarA and/or DarB have functions related to glutamate metabolism, and that the deletions of either of the corresponding genes already covers the aspect of glutamate homeostasis. Since most of the suppressors derived from the Δdac ΔdarB mutants also acquired additional mutations inactivating DarA, it seems likely that DarA is involved in controlling glutamate metabolism. We have added this to the discussion (see l. 639-651).

5. The authors alluded to the essentiality of c-di-AMP in aerobic respiration, through stepwise suppressor screens and transcriptomic analysis, and as also discussed in lines 584-598. I think some more controls should be done to establish this possible regulation and distinguish it from glutamate toxicity or other nutrient effects.

- Related to results in Table 1 and discussion point in lines 585-586: Is glutamate and potassium toxicity dependent on oxygen? Does complementation with WT ndhF restore glutamate toxicity or alter aerobic growth?

RE: This cannot be tested as the strains are not transformable. We think that oxygen affects potassium and glutamate toxicity (high proton motive force is required to fuel the major importers KimA and AimA); however, we already observed mutations related to potassium uptake under anaerobic conditions indicating the general toxicity of at least potassium irrespective of oxygen availability.

- Does the Bacillus dac mutant grow differently under aerobic and anaerobic conditions in MSSM?

RE: Both the wild type and the dac mutant grow very poorly under anaerobic conditions in MSSM (as compared to aerobic conditions), so this is not a specific issue of the dac mutant.

- Can you expand your discussion on why LB might be more permissive to anaerobic growth than aerobic growth, at least for the dac darA/B mutants?

RE: We think that aerobic respiration gives rise to a high proton motive force which is required for potassium uptake. Therefore, adaptation to LB is easier under anaerobic conditions when the major potassium transporter KimA, a proton:potassium symporter, is less active. Indeed, in the first step of adaptation (anaerobic conditions) we see mutations in KtrC in two of three mutants. KtrCD is the main potassium transporter under this condition (no need for particularly high proton motive force, stimulated by glutamate). Then, when oxygen comes into play in the second selection step, most of the mutants acquire mutations in KimA indicating that potassium uptake via KimA is a major problem for the dac mutant in the presence of oxygen.

This is also described in the discussion: “Although it is not yet understood how precisely respiration interferes with the growth of the Δdac mutant, it is tempting to speculate that the proton motive force generated by respiration plays a key role: the major potassium transporter KimA is actually a proton:potassium symporter [85], and potassium uptake by the KtrAB and KtrCD channels is directly driven by the proton motive force [86]. Thus, a reduced respiration lowers the proton motive force and may thus limit the uptake of potassium, the major growth-limiting factor for the B. subtilis Δdac mutant.” (l. 632 - 637).

- Based on the decreased expression of both ldh-lctP and cydABCD, what conclusions can be drawn about aerobic respiration vs fermentation activities in dac?

RE: This is a good point! Both operons are subject to transcription repression by Rex, and the physiological scenario of having both activities regulated in the same direction has not yet been investigated.

6. The observation that mutations in aimA were frequently isolated in the dac suppressor screen, as well as data from Figure S8 (dac aimA grew but dac gltT did not), might also indicate that the dac mutant is sensitive to serine. Have the authors examined this possibility and discerned it from glutamate toxicity?

RE: Indeed, the B. subtilis wild type strain 168 is sensitive to serine, and this is likely also the case for the dac mutant. We have just published a detailed analysis on serine sensitivity in B. subtilis, and this resulted in the identification of YbeC/ AimA as the major serine transporter (Klewing et al., 2020, Environ. Microbiol.). However, in the present study, no serine was included in the medium so that effects of serine toxicity are unlikely to play a role in our selective scenario.

Minor comments:

7. The authors should consider showing schematic diagrams of relevant pathways to better predict the mechanisms of suppressor phenotypes. For instance, an abbreviated schematic diagram of phospholipid biosynthesis showing PlsC and AccA-C would greatly clarify data interpretation in lines 367-390. An overall model figure summarizing the mechanisms of glutamate toxicity and c-di-AMP essentiality in LB would also be a fantastic addition.

RE: We have included a scheme of lipid biosynthesis as supplemental Figure S6. Moreover, we have added Fig. 6 to summarize the findings on glutamate toxicity.

8. Was gene expression in the transcriptomic data set normalized to a housekeeping gene?

RE: No, these are absolute values.

9. Section on LB suppressor screen (lines 214-277) is perhaps best moved to the end of the Results since all other sections address glutamate toxicity.

RE: We prefer to have all the unbiased analyses (both suppressor screens and transcriptome amalysis) at the beginning, and then to present the individual components in the system.

**Reviewer #3:**

This manuscript describes the isolation and analysis of suppressor mutants of a Bacillus subtilis mutant devoid of all three diadenylate cyclase genes. Whilst suppressors were unable to be obtained on rich media (unless darA or darB were also inactivated and incubation was under anaerobic conditions), several suppressors could be isolated on minimal media containing glutamate. Genome analysis revealed multiple mutations in most suppressors, with most occurring in genes encoding a novel glutamate transporter (YbeC/AimA), several different potassium transporters, phospholipid biosynthesis genes and a mechanosensitive exporter (YfkC). Further characterization revealed that AimA is a low affinity glutamate transporter while GltT is a high affinity glutamate transporter. Work also showed that potassium levels in the media affect glutamate transport by GltT (but not AimA), which would allow balanced regulation of these major ions in the cell. In addition, expression of AimA is increased in the absence of c-di-AMP, which provides an explanation of why the diadenylate cyclase mutant is sensitive to glutamate (however the mechanism of this regulation was not explored). Overall the work is interesting and provides several new insights into why c-di-AMP is essential for bacteria. The paper is clearly written and the complex findings in the suppressors are well presented.
Limitations of the work include where it describes several suppressors with mutations in genes affecting phospholipid biosynthesis and a mechanosensitive channel, however their roles in restoring growth in mutants unable to synthesize c-di-AMP is not clear. There is quite a lot of speculation that the YfkC mutation is a gain-of-function change and may lead to enhanced glutamate export, however this was not explored more. This would make sense and would be a novel finding and in alignment with the theme of the paper, which is focussed primarily on glutamate homeostasis. To strengthen the paper it would be worthwhile to determine if intracellular glutamate levels are reduced in cells carrying the variant YfkC protein (this could perhaps be performed in the wild-type strain carrying the yfkC variant).

RE: First of all, we thank the reviewer for the positive comments on our work. This reviewer sees a major limitation of this study in the explanation of mutations affecting lipid biosynthesis and the mechanosensitive channel YfkC. We agree, and therefore we have included new experimental data. In detail, we have constructed a yfkC* plsC* double mutant (please remember that plsC* alone is not tolerated by the wild type strain, see above, second major point of reviewer #1), and compared the intra- and extracellular cellular glutamate concentrations for a set of strains (wild type, ΔyfkC, yfkC*, yfkC* plsC*). These data are shown in the new Figures 4B and 4C, and they confirm our hypothesis that the combination of the yfkC* and plsC* mutations results in a decreased intracellular and increased extracellular glutamate concentration caused by increased glutamate export. We have added a new section on this topic in the Results part (see l. 410 - 432).

Specific comments:

- Line 120 – should glutamate be glutamine?

RE: Thank you, this has been corrected.

- Table 1 – should define the criteria for the +++, ++, + and – scores (is it simply colony size?)

RE: This is defined according to the dilution step that still allows growth. We have made a statement in Footnote 1 of the Table, and together with Fig. S1 this will be immediately obvious.

- Line 189 and after – change YbeC to AimA (since it was renamed in line 140)

RE: Actually, it is renamed much later in the Results section. We keep using YbeC until then. L. 140 (now 151) is rather an announcement in the Introduction.

- Lines 402-404 – the reference to Figure 4 here doesn’t strongly support the statement (the rapid appearance of suppressors in the nhaK background strain). I can see one colony on the 10E-1 dilution, but none on the 10E0 dilution, suggesting that suppressors are still relatively uncommon. I suggest to remove the reference to this figure in this sentence (or provide some stronger support from an additional experiment)

RE: We agree and have removed the reference to Fig. 4.

- Line 575 – speculation of the gain-of-function change in YfkC is not strongly supported by data.

RE: We agree and have included new experimental data on the intra- and extracellular glutamate concentrations which perfectly support our hypothesis (see Fig. 4B). We have also added a new section on this topic in the Results part (see l. 410 - 432).

- Figure 1 – there appear to be some listed mutations missing for the suppressors (GP3102 and GP3103) of the delta-dac/delta-aimA mutant.

RE: This has been corrected.
